# Supplementary material for: Zebularine, a DNA Methylation Inhibitor, Activates Anthocyanin Accumulation in Grapevine Cells
Source: Genes (Basel). 2022 Jul 15;13(7):1256. doi: 10.3390/genes13071256 (PMC9316115; doi:10.3390/genes13071256)
Supplement: Supplementary file 1 [file genes-13-01256-s001.zip › genes-1800559-supplementary.pdf]

Table S1: Genes related to the general phenylpropanoid pathway, the flavonoid biosynthetic pathways, and the anthocyanin specific pathway.

Name	Enzymatic activity / transporter type	Gene number	References	Gene IDs
**General phenylpropanoid biosynthetic genes**				
PAL	phenylalanine ammonia lyase	15	[6][3]	Vitvi00g01367; Vitvi06g00256; Vitvi08g01022; Vitvi13g00622; Vitvi11g00126; Vitvi11g00116; Vitvi16g00066; Vitvi16g01507; Vitvi16g01502; Vitvi16g00054; Vitvi16g00061; Vitvi16g01503; Vitvi16g00060; Vitvi16g00055; Vitvi16g00057
C4H	cinnamate-4-hydroxylase	3	[7][1]	Vitvi11g00924; Vitvi11g01045**; Vitvi06g00803**
4CL	4-coumarate-coA ligase	14		Vitvi01g01561; Vitvi02g00938; Vitvi06g01318; Vitvi08g01625; Vitvi11g01257; Vitvi11g01258; Vitvi13g00701; Vitvi14g01588; Vitvi14g01589; Vitvi16g00139; Vitvi17g00148; Vitvi18g00126; Vitvi18g00124; Vitvi01g01661
**Flavonoid-related genes**				
CHS	chalcone synthase	3	[6][3]; [9][2]	Vitvi14g01449; Vitvi14g01448; Vitvi05g01044
CHI	chalcone isomerase	6	[6][3]	Vitvi13g00225; Vitvi13g01911; Vitvi19g00009; Vitvi14g01683; Vitvi07g00128; Vitvi04g00175
F3'5'H	flavonoid 3′,5′-hydroxylase	5	[11][1]; [17][2]	Vitvi06g01192; Vitvi06g01888; **Vitvi06g01885**; Vitvi06g01206; Vitvi08g01637
F3'H	flavonoid 3′-hydroxylase	2	[11][1]; [17][2]	Vitvi17g00698; **Vitvi17g00700**
F3H	flavanone-3-hydroxylase	2	[6][3]	Vitvi04g01454; Vitvi18g01119
DFR	dihydroflavanol reductase	1	[6][3]; [13][1]	**Vitvi18g00988**
LDOX	Leucoanthocyanidin dioxygenase	1	[6][3]	Vitvi02g00435
**Genes specific for anthocyanin biosynthesis**				
UFGT	UDP Glc:flavonoid 3-O-glucosyltransferase	1	[6][3]; [8][1]	**Vitvi16g00156**
AOMT	Anthocyanin O-methyltransferase	4	[16][1]; [18][1]; [19][1]	**Vitvi01g02265; Vitvi01g02263;** Vitvi01g01635; Vitvi01g02261
AT	Acyltransferases	2	[23][1]	**Vitvi03g01816**; Vitvi03g00077
**Genes involved in the transport of anthocyanins into the vacuole**				
GST	glutathione S-transferase	5	[14][1]; [24][1]	Vitvi19g01328; Vitvi19g02197; Vitvi19g01338; Vitvi04g00880; Vitvi04g00884
AM	Transporters (Multidrug And Toxic Extrusion family)	3	[15][1], [20][1]; [22][3]	**Vitvi16g01911**; **Vitvi16g01915;** Vitvi16g01913
ABCC	Transporters (ATP binding cassette protein)	1	[21][1]	**Vitvi16g01210**

For each enzyme or transporter, the genes were selected according to the bibliography (see references mentioned in the table), and completed with a search for the closest paralogous genes, using Ensembl Plant (https://plants.ensembl.org/index.html), with identity percentage between the two genes > 50%, except for CHS genes and genes specifically related to anthocyanin biosynthesis and transport, for which only genes with identity percentage >75% were selected).

[1]: functional characterization (the gene which were characterized are in bold letters); [2]: analysis of the gene family; [3]: cloning.

Table S2: genes identified as regulators of anthocyanin, proanthocyanidin, or flavonol biosynthesis

Name	Biosynthetic pathways	Activity	Gene ID	Target genes	References
MYBA1	Anthocyanins	Activator	Vitvi02g01019	CHI3, F3'5'H, UFGT, 3AT, GST4, OMT	[23]; [27]; [30]; [31]; [33]; [34]
MYBA2	Anthocyanins	Activator	Vitvi02g01015	UFGT	[26]; [28]; [29]
MYBA5	Anthocyanins	Activator	Vitvi14g00940	UFGT, 3AT	[33]
MYBA6	Anthocyanins	Activator	Vitvi14g00930	UFGT, 3AT	[33]
MYBA7	Anthocyanins	Activator	Vitvi14g00925	UFGT, 3AT	[33]
MYBC2-L1	anthocyanins + proanthocyanidins	Repressor	Vitvi01g00401	UFGT, GST4, OMT	[32], [34]
MYBPA1	Early flavonoid and proanthocyanidins	Activator	Vitvi15g00938	CHS3, CHI, F3'5'H, LAR1, LAR2, LDOX, ANR	[26]; [28]; [29]; [31]; [33]
MYBPA2	proanthocyanidins	Activator	Vitvi11g00099	F3'5'H, LAR1, LAR2, ANR	[31]
MYBPAR	proanthocyanidins	Activator	Vitvi11g00097	F3'5'H, LAR1, LAR2, ANR	[31]
MYBF1	Early flavonoid biosynthetic pathway + flavonols	Activator	Vitvi07g00393	CHI, FLS1	[28]
MYB13	Stilbenes	Activator	Vitvi05g01732		
MYB14	Stilbenes	Activator	Vitvi07g00598	STS29, STS41	[88]
MYB15	Stibenes	Activator	Vitvi05g01733	STS29, STS41	[88]

Only references presenting experimental demonstration of the identity of the direct target genes are included.

RT-qPCR		
Gene	Primer	5'-3' sequence
VvEF1	EF1-F	CAAGAGAAACCATCCCTAGCTG
	EF1-R	TCAATCTGTCTAGGAAAGGAAG
VvMYBA1	VvMYBA1-F	AAGCCATCATCCACTTCACC
	VvMYBA1-R	TCTCTCCAGAAGCCGAAAAG
VvMYBA2	VvMYBA2-F	AGACTCGATGAAGAGCTTAGG
	VvMYBA2-R	CTTTAGGCATCTATTCAACC
VvMYC1	MYC-F	GGAAGTAAAGAGGGCAATAAA
	MYC-R	CTACAAACATCAGCAACAATACCATA
VvCHI	CHI-F	AGGAGTTAGCGGATTCGGTTGAC
	CHI-R	AAGGCAACACAATTCTCTGACACC

McrBC-qPCR		
Targeted sequence	Primer	5'-3' sequence
prActin	VvprActin-F1	TCTCACTAGCTCACTCAACC
	VvprActin-R2	CAGCCGACTCTGATAACTCC
retrotransposon GRET1	VvGret1LTR1-F1	AGCAAGTCTTACACGCTATCAC
	VvGret1LTR1-R1	TAGAATGATCGTGGGAAGTGAC
retrotransposon GRET1	VvGret1LTR1-F2	GACAACAAGTCACTTCCCAC
	VvGret1LTR1-R2	ATGATGACATTCGGAGGCAG
retrotransposon CACTA	VvCACTA-F1	TGTTGTCACTACTTATTCCTGGTC
	VvCACTA-R1	CGTCCCATAAGTCATTCAACTC
retrotransposon CACTA	VvCACTA-F2	AAACTTCGATAACGTACACCC
	VvCACTA-R2	CCACATGCTGTATGAAATTGAC
prUFGT	VvprUFGT-F3	AAACAGTTATCAAACAGACCT
	VvprUFGT-R3	TTTCGTCATTTCATCTTGG
prMYBA2	VvprMYBA2-F3	AAATTCTAGTTGTAAGACTGTAGAC
	VvprMYBA2-R5	AAGAAATAACAACTCTCACGG

Table S3: list of the primers used for RT-qPCR and McrBC-PCR

A

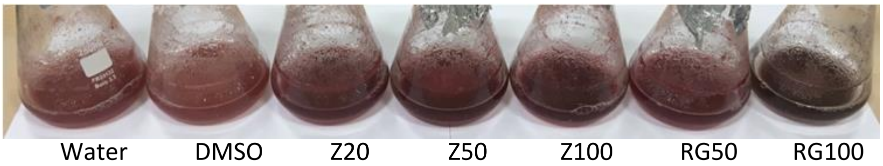

B

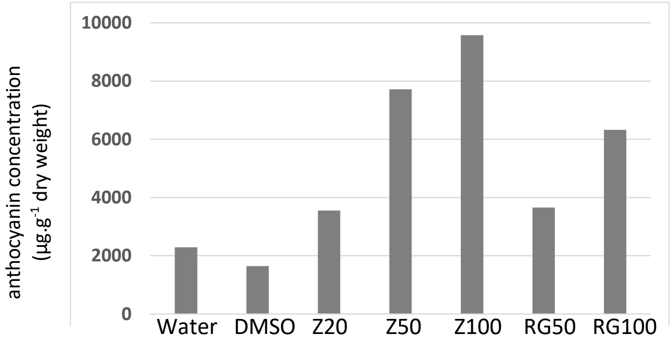

C

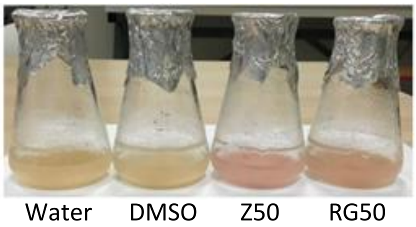

D

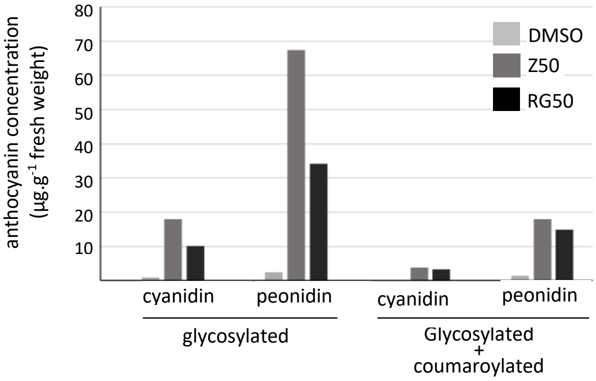

Figure S1: **Two DNA methyltransferase inhibitors–RG108 and zebularine - stimulate anthocyanin biosynthesis in grape cell cultures.** Treatments with zebularine and RG108 were performed 3 days after sub-culturing. The MS medium was supplemented with zebularine, RG108 or DMSO, which was used as a solvent for zebularine and RG108. An additional control with water was included. The experiment was performed in the light **(A, B)** with zebularine 20 µM (Z20), 50 µM (Z50) or 100 µM (Z100) and RG108 50µM (RG50) or 100µM (RG100), and in the dark **(C, D)**, with zebularine 50µM (Z50) and RG108 50µM (RG50). Ten days after sub-culturing, cell suspensions were photographed **(A, C)** and harvested for anthocyanin quantification **(B, D)**. The concentration of individual anthocyanin was determined as described in the Material and methods. In B, cell total anthocyanin content was calculated as the sum of each individual anthocyanin. This experiment was performed only once, therefore no statistical analysis is available

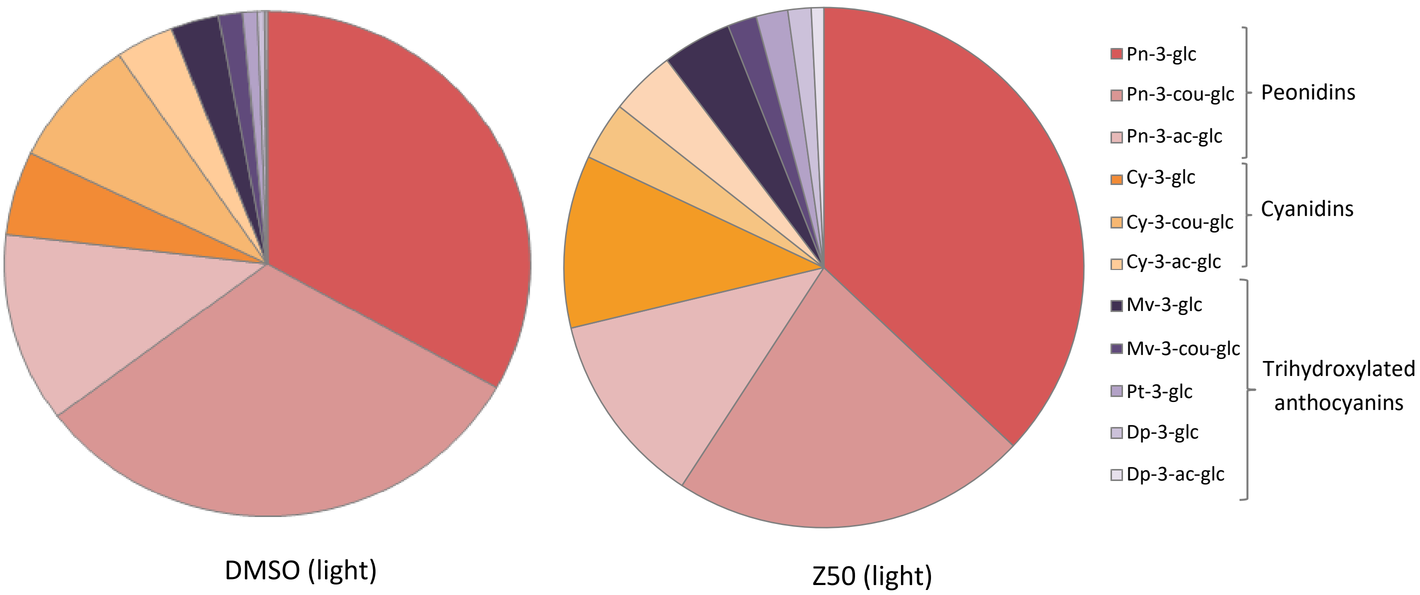

Figure S2. Zebularine does not significantly affect anthocyanin composition. The two chart pies represent the proportions of the different anthocyanins in light grown GT cells 12 days after subculture, in control conditions (DMSO) and after a treatment with zebularine 50µM (Z50). The concentration of individual anthocyanin was determined in three independent biological replicates, and the proportion of each individual anthocyanin was calculated using the mean of the concentrations of the three replicates. Pn-3-glc, peonidin-3-glucoside; Pn-3-cou-glc, peonidin-*3*-(6''-*p-coumaroyl-glucoside)*; Pn-3-ac-glc, peonidin-3-(6''-acetylglucoside); Cy-glc, cyanidin-3-glucoside; Cy-3-cou-glc, cyanidin-*3*-(6''-*p-coumaroyl-glucoside)*; Cy-3-ac-glc, cyanidin-3-(6''-acetylglucoside); Mv-3-glc, malvidin-3-glucoside; Mv-3-cou-glc, malvidin-*3*-(6''-*p-coumaroyl-glucoside)*; Pt-3-glc, petunidin-3-glucoside; Dp-3-glc, delphinidin-3-glucoside; Dp-3-ac-glc, delphinidin-3-(6''-acetylglucoside).

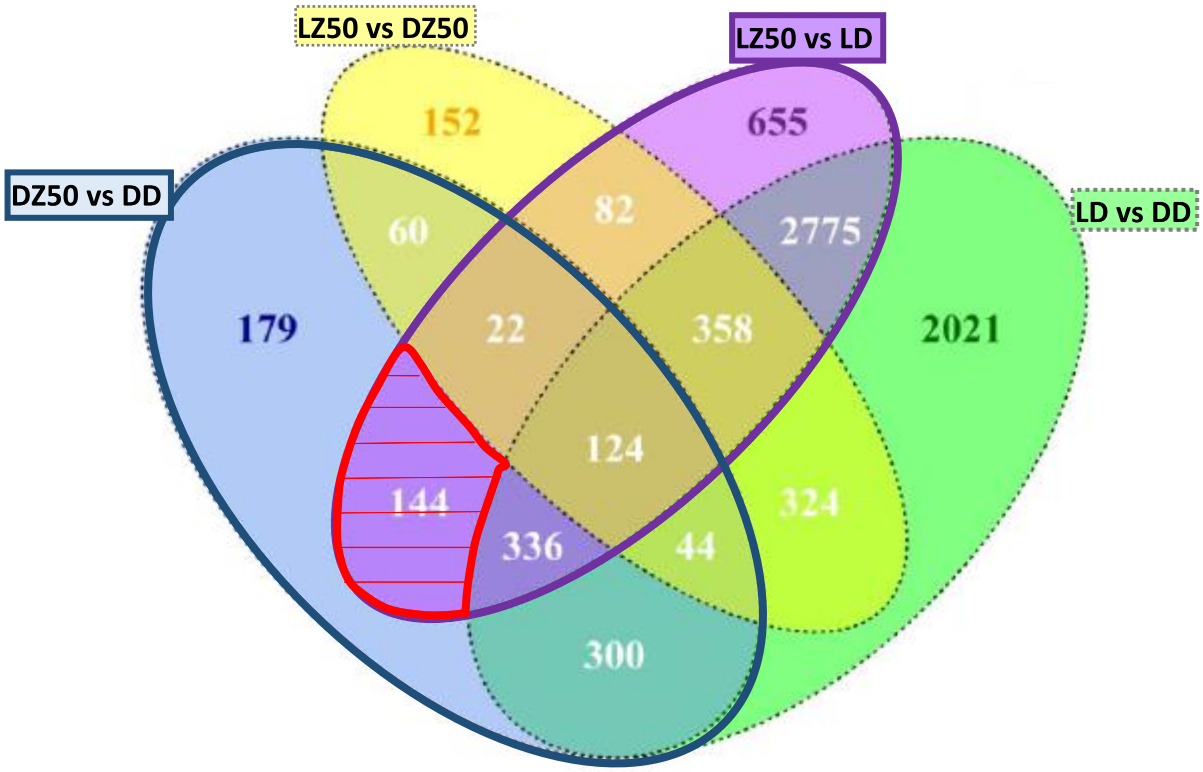

Figure S3: Only 144 genes were specifically identified as zebularine-dependent DEGs. Venn diagram displaying the number of DEGs identified for each pairwise comparison. The number of DEGs commonly identified in 2 different pairwise comparisons are indicated. The 144 genes which were identified as DEGs when comparing control and zebularine-treated cells both in light and dark, and in no other comparison are indicated by a red shape.

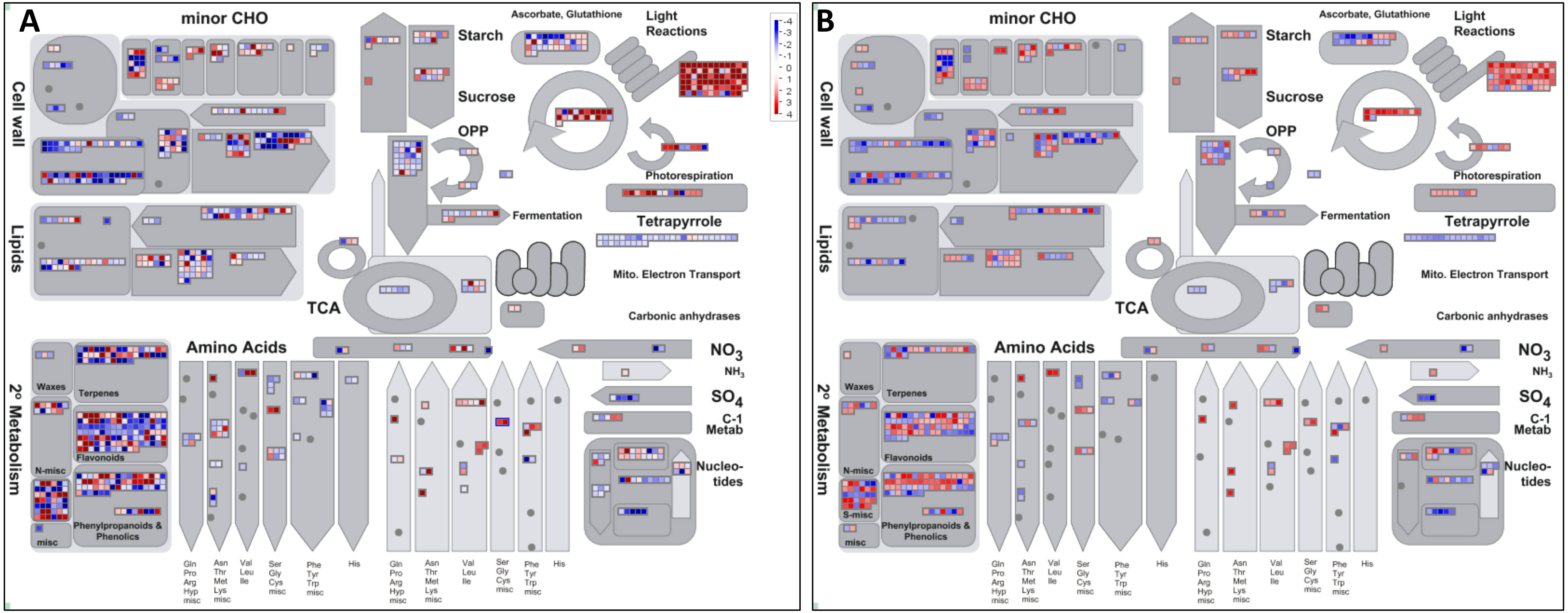

Figure S4. MapMan analyses revealed a high similarity between the genes which were deregulated by light in the control cells and the genes which were deregulated by zebularine in the light. Overviews of differences in expression of genes involved in primary and secondary metabolisms. Heat maps show genes with statistically significant (P value < 0.05) differential expression identified by comparing LD to DD cells (**A**) and LD to LZ50 cells (**B**). A conventional red-to-blue scale was used to indicate up-regulation (red) or down-regulation (blue) by light. Hence in A, red boxes indicate genes which are up-regulated in LD compared to DD, whereas in B, they indicate genes which are up-regulated in LD compared to LZ50.

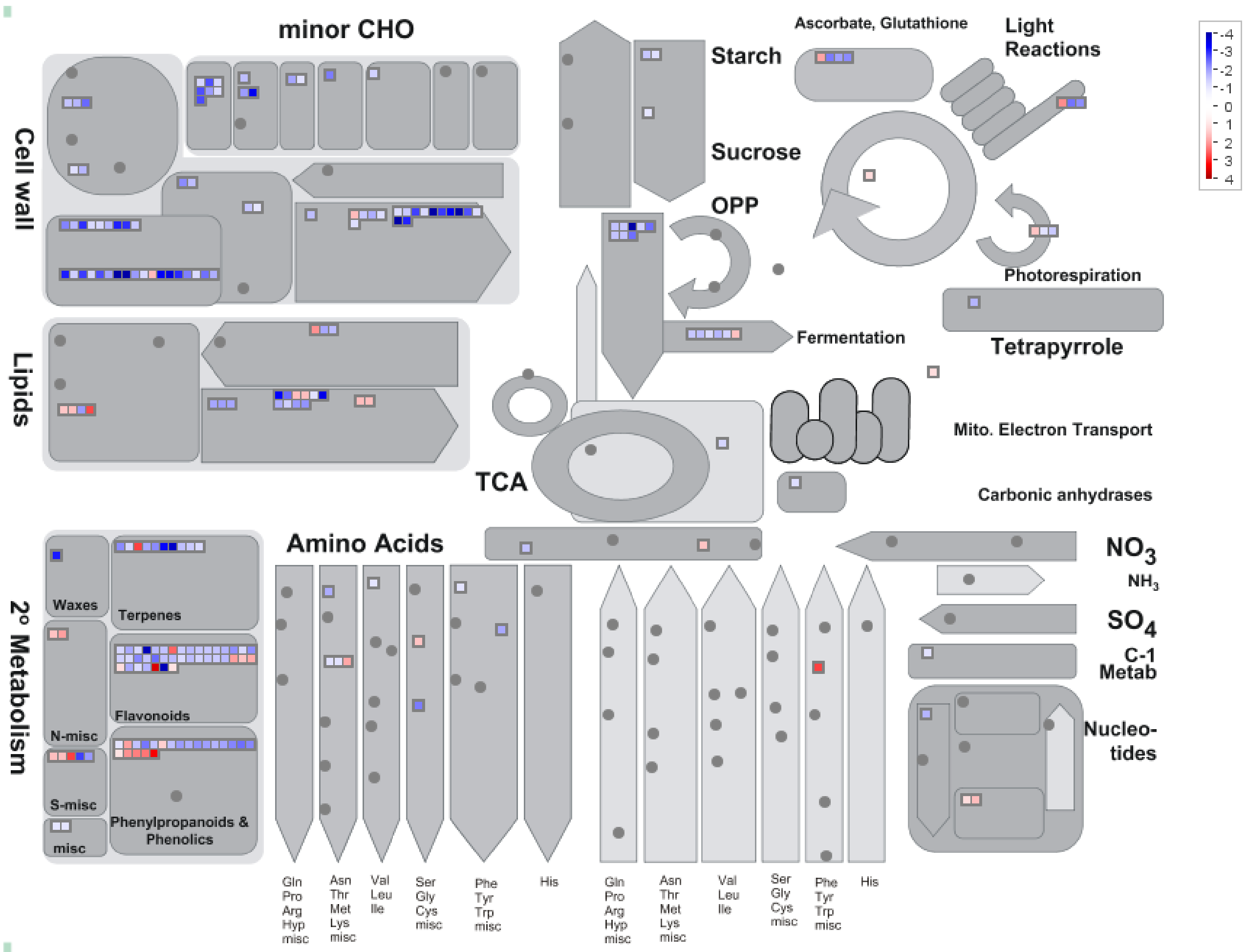

Figure S5. A MAPMAN analysis revealed a global transcriptional repression of metabolism-related genes by zebularine in the dark. Overview of differences in expression of genes involved in primary and secondary metabolisms. Heat maps show genes with statistically significant (P value < 0.05) differential expression identified by comparing DZ50 to DD cells. A conventional red-to-blue scale was used to indicate up-regulation (red) or down-regulation (blue) by zebularine. Hence red boxes indicate genes which are up-regulated in DZ50 compared to DD.

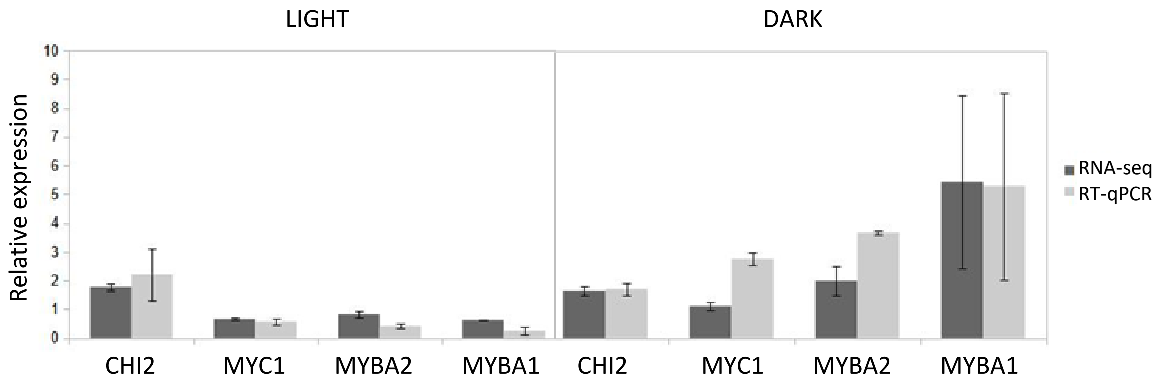

Figure S6: Comparison of RNA-seq and RT-qPCR expression data for 4 different genes, CHI (Vitvi13g00225), MYC1 (Vitvi07g00139), MYBA2 (Vitvi02g01015) and MYBA1 (Vitvi02g01019). For each gene the expression in zebularine treated cells (50µM) was expressed comparatively to the expression in DMSO treated cells. The relative expression was calculated for each of the 3 biological replicates, and the mean of the three relative expression values was reported on the graph. Error bars indicate standard deviation (SD) for the three replicates. For RT-qPCR quantification, EF1α was used as a reference gene.

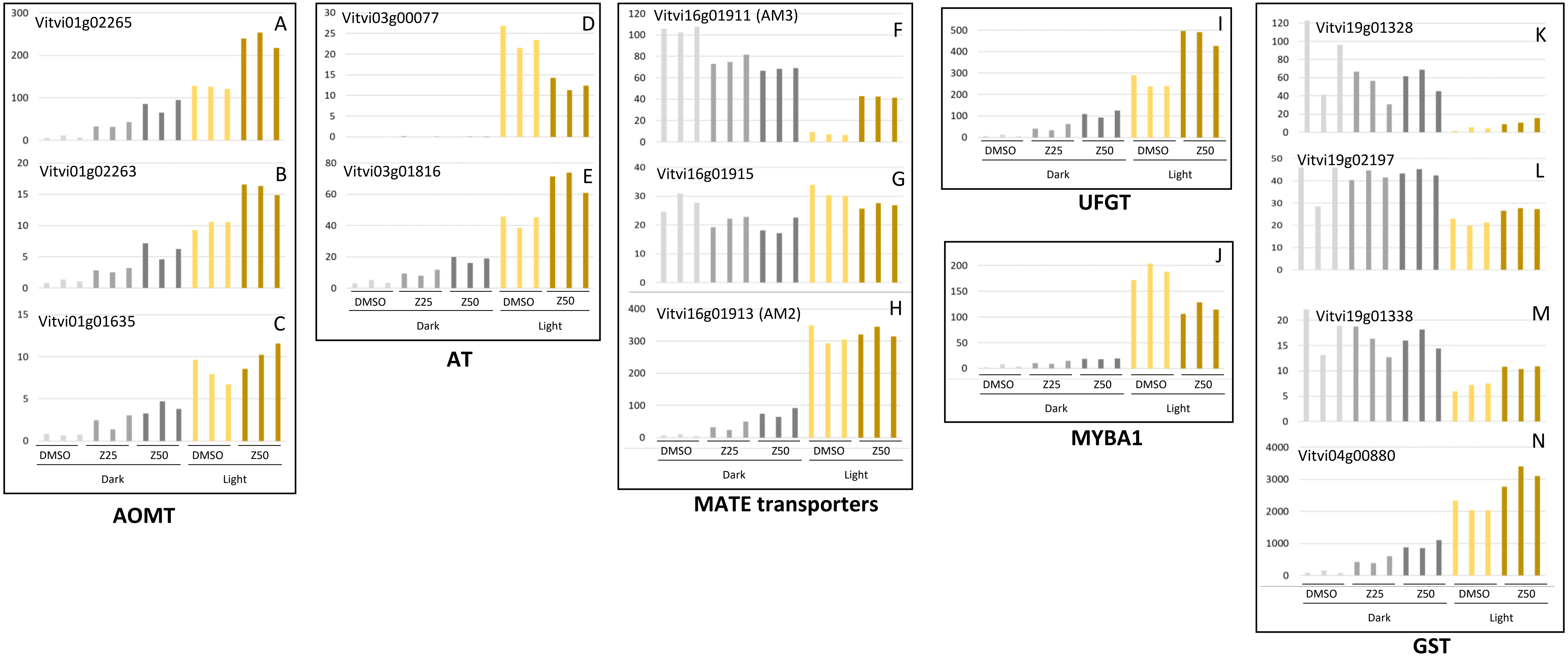

Figure S7: RNA-seq results for a few selected anthocyanin-related genes. The results are expressed in reads per kilo base per million mapped reads (rpkm). For each growth condition, three results are shown corresponding to three different biological replicates. For dark and light grown cells, different treatments were compared: DMSO, zebularine 25µM (Z25) or zebularine 50µM (Z50). **A**. AOMT - Vitvi01g02265; **B**. AOMT - Vitvi01g02263; **C**. AOMT - Vitvi01g01635; **D**. AT - Vitvi03g00077; **E**. AT - Vitvi03g01816; **F**. MATE transporter - Vitvi16g01911; **G**. MATE transporter - Vitvi16g01915; **H**. MATE transporter - Vitvi16g01913; **I**. UFGT - Vitvi16g00156; **J**. MYBA1. Vitvi02g01019; **K**. GST - Vitvi19g01328; **L**. GST - Vitvi19g02197; **M**. GST - Vitvi19g01338; **N**. GST - Vitvi04g00880.
Vitvi01g02261 coding for an AOMT and Vitvi04g00884 coding for a GST were not included in this figure because their expression was respectively very low (< 2,5 rpkm in all samples) and undetectable in this study.

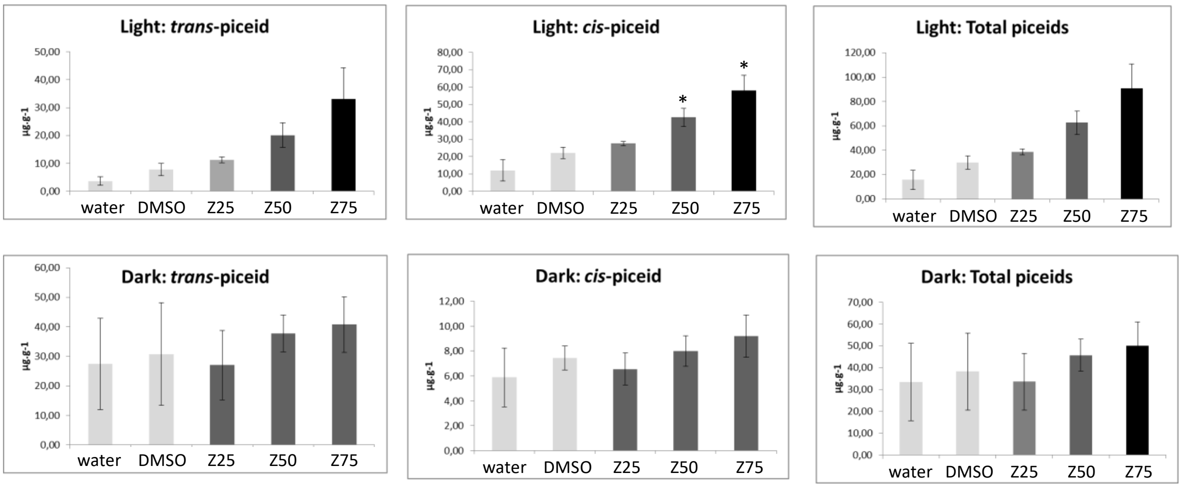

Figure S8: Stilbene accumulation (µg g^{-1} FW) in GT cell suspensions supplied with zebularine. Values represent the mean +/- SD of triplicate assays. Welch t-test was used to analyze the difference between the means of zebularine-treated and DMSO-treated samples. One star (*) was assigned to zebularine-treated samples when their polyphenol content differs significantly from the DMSO-treated samples (pvalues < 0.05). Resveratrol was detected in too low amounts to be precisely quantified.

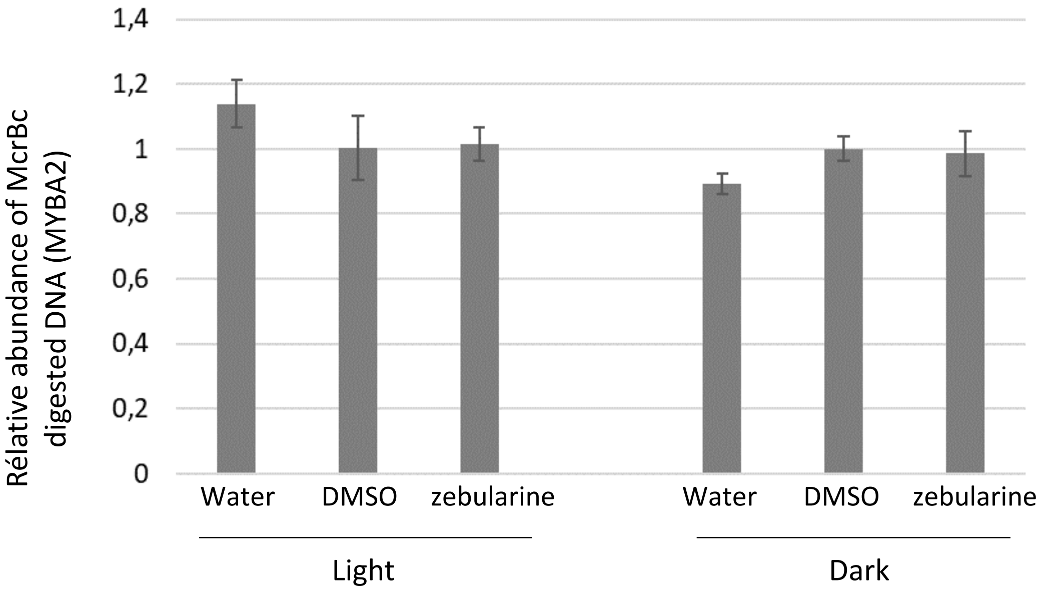

Figure S9: No variation in DNA methylation was detected in the promoter of MYBA2 in zebularine-treated cells. The methylation level was measured by McrBC-qPCR at a sequence located in MYBA2 5'region, 1200 bp upstream from the ATG. The relative abundances of the amplicons in the different McrBC-treated samples (normalization to ACTIN and to DMSO-treated samples) are shown. Bars represent the mean values for three to seven biological replicates with their standard deviations. Three biological replicates were used for all water- and DMSO-treated cells, five for the dark grown zebularine-treated cells (two replicates treated with 25µM, one with 50µM and one with 75µM), and seven for the light grown zebularine-treated cells (three replicates treated with 25µM, two with 50µM and two with 75µM).

A

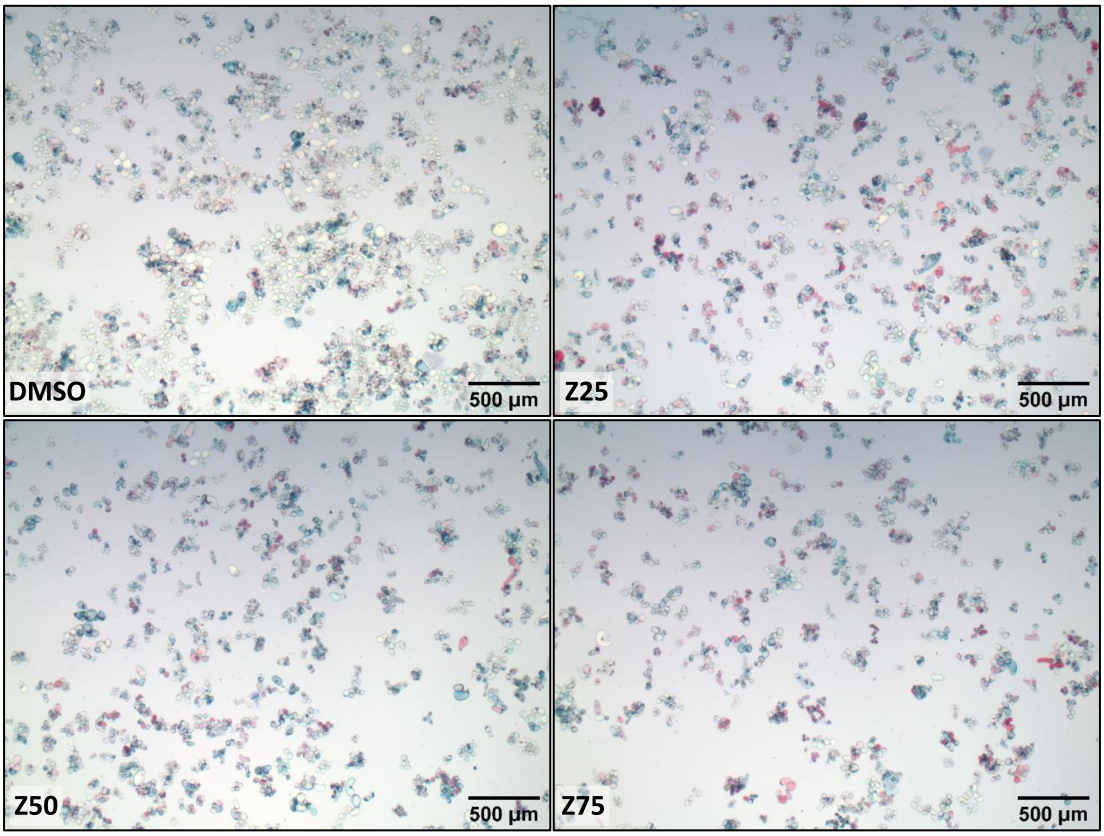

B

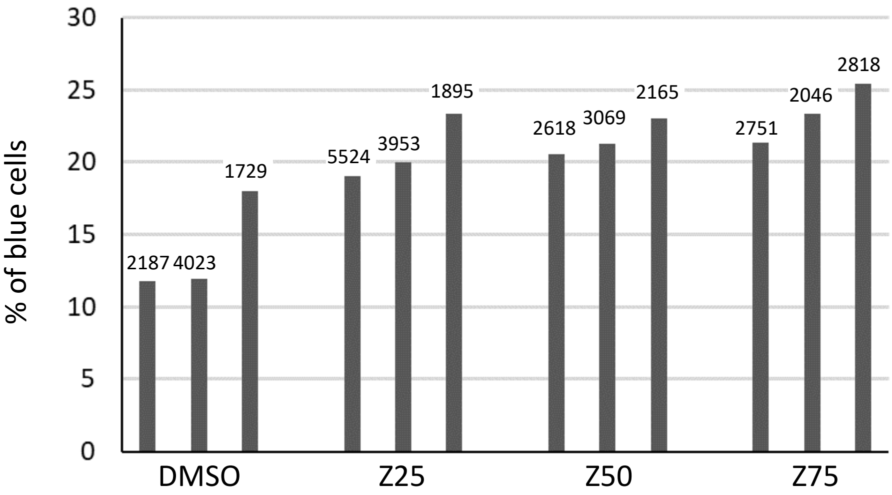

Figure S10: **Zebularine affects GT cell viability.** Cells were grown in the light. Treatments with zebularine were initiated 3 days after sub-culturing. Aliquots of different cell suspensions were collected 7 days after zebularine addition and observed by light microscope after trypan blue staining. (**A)** shows four representative pictures corresponding to the four different culture conditions. (**B**) The percentage of blue cells was quantified by manual counting, using 3 pictures corresponding to 3 biological replicates for each condition. The total number of cells counted for each analyses is indicated on top of the bars.

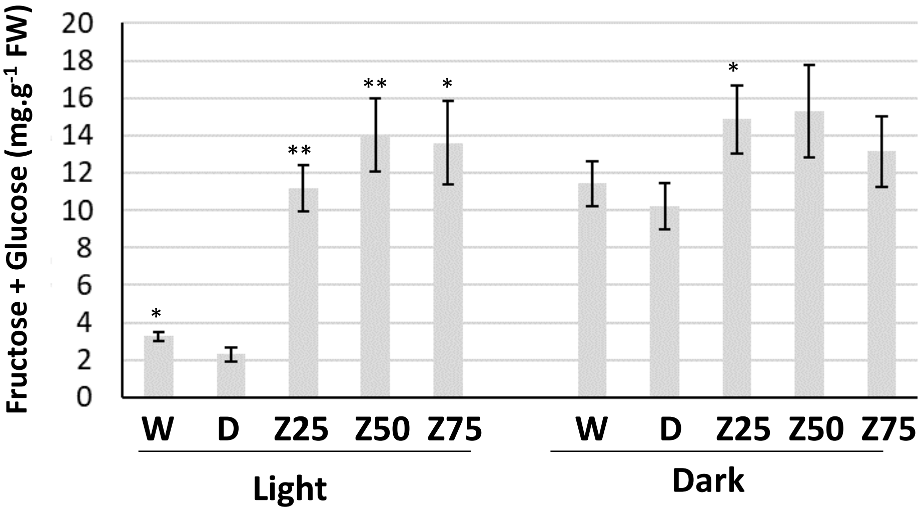

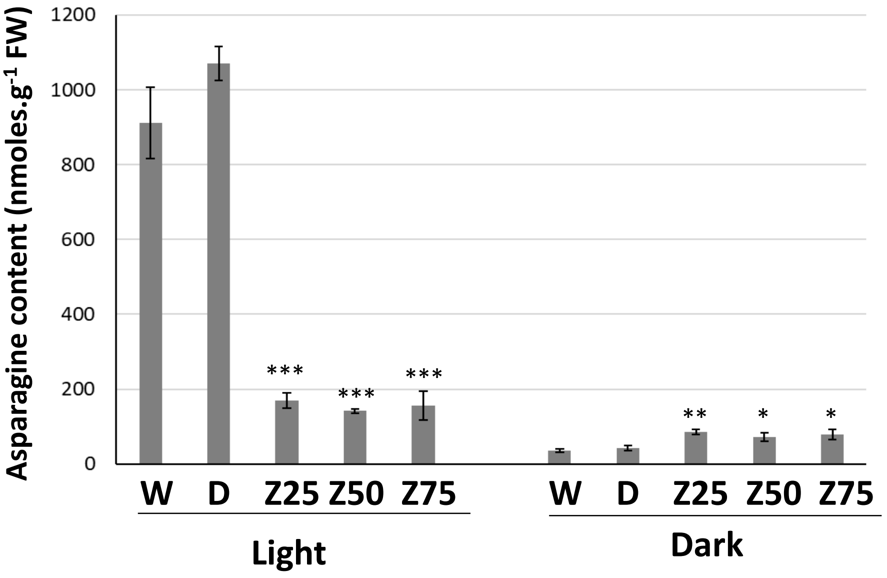

Figure S11: Control (DMSO) light grown cells present hallmarks of carbon starvation, with very low sugar content (A) and important accumulation of asparagine (B). Values are the mean ± SD of three biological replicates. Asterisks indicate significant differences in the total amounts of glucose and fructose, as determined by a Welsh's t-test *(n = 3)* based on the mean differences between zebularine-treated and DMSO-treated samples (*P < 0.05; **P < 0.01; ***<0,001).

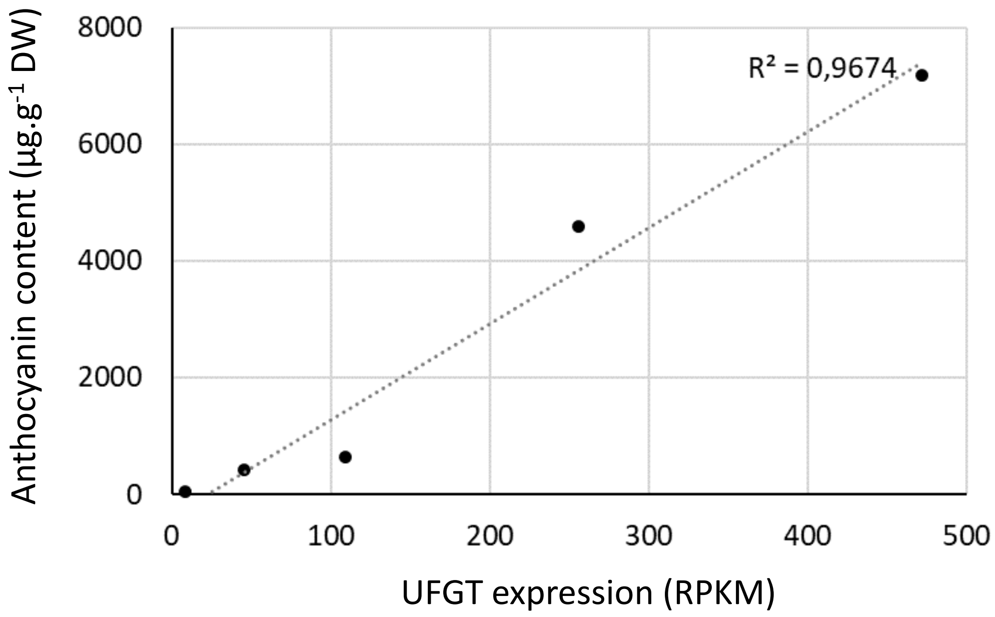

Figure S12: The *UFGT* gene expression is highly correlated with anthocyanin content. The graph was constructed using the anthocyanin content and *UFGT* expression from 5 different growth conditions (DD, DZ25, DZ50, LD, LDZ50). For each condition, the mean of the three biological replicates were taken into account. *UFGT* expression was deduced from the RNA-seq experiment data shown in Figure S7. RPKM: Reads per kilo base per million mapped reads.
